# Efficacy and safety of new anti-CD20 monoclonal antibodies versus rituximab for induction therapy of CD20^+^ B-cell non-Hodgkin lymphomas: a systematic review and meta-analysis

**DOI:** 10.1038/s41598-021-82841-w

**Published:** 2021-02-05

**Authors:** Chengxin Luo, Guixian Wu, Xiangtao Huang, Yanni Ma, Yali Zhang, Qiuyue Song, Mingling Xie, Yanni Sun, Yarui Huang, Zhen Huang, Yu Hou, Shuangnian Xu, Jieping Chen, Xi Li

**Affiliations:** 1 Center for Hematology, Southwest Hospital, Third Military Medical University, Chongqing, China; 2 Key Laboratory of Cancer Immunotherapy of Chongqing, Southwest Hospital, Third Military Medical University, Chongqing, China; 3 Department of Health Statistics, Third Military Medical University, Chongqing, China; 4 Institute of Infectious Disease, Southwest Hospital, Third Military Medical University, Chongqing, China

**Keywords:** B-cell lymphoma, B-cell lymphoma

## Abstract

Rituximab combined with chemotherapy is the first-line induction therapy of CD20 positive B-cell non-Hodgkin lymphomas (CD20^+^ B-NHL). Recently new anti-CD20 monoclonal antibodies (mAbs) have been developed, but their efficacy and safety compared with rituximab are still controversial. We searched MEDLINE, Embase, and Cochrane Library for eligible randomized controlled trials (RCTs) that compared new anti-CD20 mAbs with rituximab in induction therapy of B-NHL. The primary outcomes are progression-free survival (PFS) and overall survival (OS), additional outcomes include event-free survival (EFS), disease-free survival (DFS), overall response rate (ORR), complete response rate (CRR) and incidences of adverse events (AEs). Time-to-event data were pooled as hazard ratios (HRs) using the generic inverse-variance method and dichotomous outcomes were pooled as odds ratios (ORs) using the Mantel–Haenszel method with their respective 95% confidence interval (CI). Eleven RCTs comprising 5261 patients with CD20^+^ B-NHL were included. Compared with rituximab, obinutuzumab significantly prolonged PFS (HR 0.84, 95% CI 0.73–0.96, *P* = 0.01), had no improvement on OS, ORR, and CRR, but increased the incidences of serious AEs (OR 1.29, 95% CI 1.13–1.48, *P* < 0.001). Ofatumumab was inferior to rituximab in consideration of ORR (OR 0.73, 95% CI 0.55–0.96, *P* = 0.02), and had no significant differences with rituximab in regard to PFS, OS and CRR. ^131^I-tositumomab yielded similar PFS, OS, ORR and CRR with rituximab. ^90^Y-ibritumomab tiuxetan increased ORR (OR 3.07, 95% CI 1.47–6.43, *P* = 0.003), but did not improve PFS, DFS, OS and CRR compared with rituximab. In conclusion, compared with rituximab in induction therapy of CD20^+^ B-NHL, obinutuzumab significantly improves PFS but with higher incidence of AEs, ofatumumab decreases ORR, ^90^Y-ibritumomab tiuxetan increases ORR.

## Introduction

Non-Hodgkin lymphomas (NHL) are a group of heterogeneous malignant disorders arising from lymphocytes at various stages of differentiation^1^. Approximately 85–90% of NHL origin from B cells^2^. CD20 is an ideal target for therapy of B-cell non-Hodgkin lymphomas (B-NHL) due to its specific expression pattern and unique biological property^3^. Since its initial approval in 1997, the anti-CD20 monoclonal antibody rituximab has greatly improved the survival outcome of B-NHL patients with acceptable toxicity^4,5^. However, despite the success of rituximab in the treatment of CD20^+^ B-NHL, there are patients that fail to respond to initial therapy or relapse sooner.

To overcome the resistance and enhance anti-tumor activities, next generations of anti-CD20 monoclonal antibodies (mAbs) were developed. The second-generation anti-CD20 mAbs are a group of fully humanized IgG1 antibodies, including ofatumumab, veltuzumab, and ocrelizumab^6^. Among them, ofatumumab is the most widely investigated. The drug is a type I anti-CD20 mAb generated via transgenic mouse and hybridoma technology^7^. The proposed advantages of ofatumumab over rituximab include enhanced binding affinity to CD20 and greater complement-dependent cytotoxicity (CDC) against target cells as verified with in vitro studies^8^. Ofatumumab has been approved for the treatment of relapsed and refractory chronic lymphocytic leukemia (CLL) by the United States Food and Drug Administration (FDA) and the European Medicines agency (EMA) in 2009 and 2010 respectively, but the efficacy of ofatumumab in patients with B-NHL is still being investigated and there is still no inconsistent result^9^. The third-generation anti-CD20 mAbs are a group of fully humanized and engineered antibodies that include obinutuzumab, ocaratuzumab and PRO131921^6^. Until now, obinutuzumab (GA101) has been approved for the treatment of CLL and follicular lymphoma (FL) by FDA and EMA^10^. Obinutuzumab is a type II anti-CD20 mAb with glycoengineered Fc region which can enhance binding affinity to the Fc receptor (FcR) on immune effector cells^8^. In vitro studies have proved that obinutuzumab has more potent direct cell death (DCD) and more effective antibody-dependent cellular cytotoxicity (ADCC) to target cells compared with rituximab^11,12^. Clinical trials which investigated whether obinutuzumab is superior to rituximab in the treatment of patients with other subtypes of B-NHL were performed, but the results are inconsistent. Additionally, novel agents that conjugate radioisotope to anti-CD20 mAbs, such as ^131^I-tositumomab (Bexxar) and ^90^Y-ibritumomab tiuxetan (Zevalin), have also been developed. The postulated advantages of these agents are that they could cause a crossfire effect and eradicate nearby tumor cells that are not targeted by antibody but are affected by radiation^14^. Promising results from clinical trials promote the approval of ^131^I-tositumomab (Bexxar) and ^90^Y-ibritumomab tiuxetan (Zevalin) by FDA for the treatment of FL in 2003 and 2002 respectively^13,14^. Although ^131^I-tositumomab (Bexxar) is now unavailable since the discontinued production by manufacturer in 2014, a number of well-designed trials have been performed to compare its efficacy with rituximab in patients with different subtypes of B-NHL before that^13,14^.

Above all, a lot of clinical trials have been performed to compare the efficacy and safety of multiple new anti-CD20 mAbs with rituximab for the treatment of B-NHL, but the results of these trials are inconsistent. It remains unclear that whether new anti-CD20 mAbs are superior to rituximab. This systematic review and meta-analysis aimed to assess the efficacy and safety of new anti-CD20 mAbs compared with rituximab in the induction therapy of CD20^+^ B-NHL.

## Methods

### Literature search and study selection

We searched MEDLINE, Embase, and Cochrane Library from inception to March 11th, 2019 with no language restriction. The search strategies for each database are presented in Supplementary Appendix. Reference lists of included trials and relevant reviews were manually checked for additional trials.

Two investigators (CXL and XL) independently assessed eligibility of citations identified by the above search. Disagreements were resolved by discussion with a third investigator (SNX). Clinical trials that met the following criteria were included: (i) patients with CD20^+^ B-NHL; (ii) randomly assigned patients to rituximab based therapy or other new anti-CD20 mAbs based therapy in induction therapy; (iii) reported data for at least one of the clinical outcomes, including progression-free survival (PFS), overall survival (OS), event-free survival (EFS), disease-free survival (DFS), overall response rate (ORR), complete response rate (CRR), and adverse events (AEs).

### Data extraction and quality assessment

Two investigators (CXL and XL) independently extracted data using predesigned data collection forms and cross-checked to reach a consensus. Data was extracted on trial characteristics, patient characteristics, dose and cycle of anti-CD20 mAbs, concomitant chemotherapy, follow-up and clinical outcomes. The hazard ratios (HRs) for survival outcomes were extracted or estimated with the methods previously established by Tierney et al*.*^15^. For trials with multiple reports for survival outcomes, we extracted data from the report with the longest follow-up. Methodological quality of each included trials was assessed based on random sequence generation, allocation concealment, blinding, incomplete outcome data, and selective outcome reporting, following the guidelines in Cochrane handbook^16^. Any disagreements were resolved by consensus.

### Statistical analyses

The primary outcomes of the meta-analysis are PFS and OS. The secondary outcomes are EFS, DFS, ORR, CRR, and incidences of AEs. The pooled HRs and 95% confidence intervals (CIs) for time-to-event data including PFS, OS, EFS and DFS were calculated using the generic inverse-variance method. The pooled odds ratios (ORs) and 95% CIs for dichotomous data including ORR, CRR and incidences of AEs were calculated using the Mantel–Haenszel method.

Statistical heterogeneity across trials was assessed by χ^2^ test with a significant level at *P* < 0.1 and quantified with *I*^2^ statistic. Fixed-effects model was adopted for summary estimation if heterogeneity was not significant; otherwise, random-effects model was adopted. Subgroup analyses were performed to assess the influences of treatment history, disease subtype, concomitant chemotherapy, and patient characteristics on the therapeutic effect of new anti-CD20 mAbs versus rituximab. Sensitivity analysis was performed by omitting trial with number of patients less than 20.

All analyses were conducted in Review Manager version 5.3 (Revman; the Cochrane Collaboration; Oxford, England). All *P* values were two sides and the threshold of significance was *P* < 0.05 except that for heterogeneity test. This work was reported according to preferred reporting items for systematic reviews and meta-analyses (PRISMA) statement^17^.

## Results

### Characteristics of included trials

**Figure 1:**
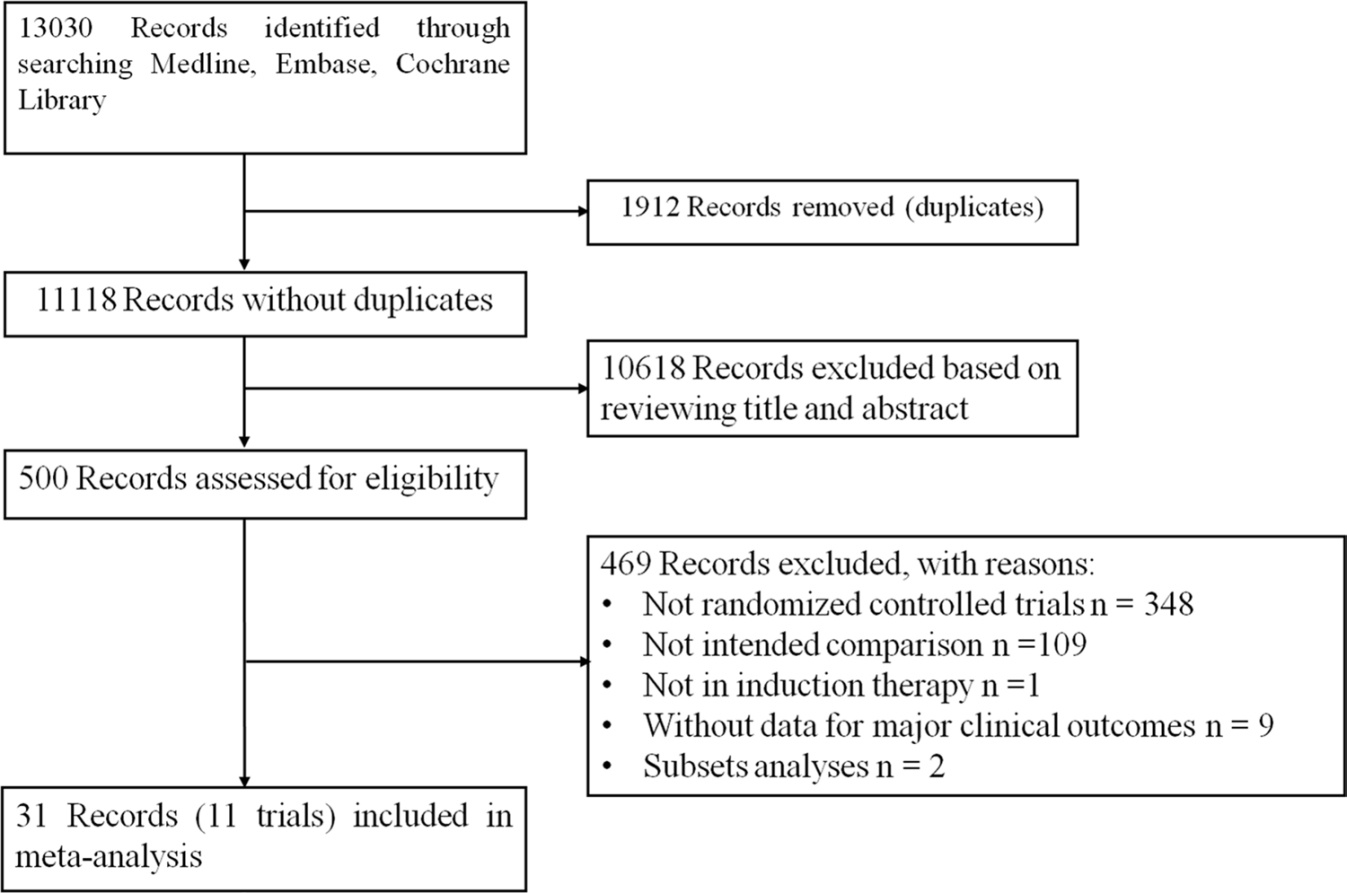
Flow chart of study selection. The PRISMA flow chart depicting study screening and selection.

Database search yielded 13,030 records. After removing 1912 duplicates, 11,118 records were screened, and 10,618 irrelevant records were excluded based on reviewing title and abstract. The remaining 500 records were retrieved as full-text publications for further evaluation. Ultimately, 11 eligible RCTs in 31 publications were included in the meta-analysis^18–32^ (Fig. 1). No additional trials were identified from the references of the included trials and the relevant reviews.

All included trials were two-arm RCTs which compared new anti-CD20 mAb with rituximab in induction therapy of CD20^+^ B-NHL. Among them, four trials compared obinutuzumab with rituximab, two compared ofatumumab with rituximab, three compared ^131^I-tositumomab with rituximab, and another two compared ^90^Y-ibritumomab tiuxetan with rituximab. No prospective RCTs compared other new anti-CD20 mAbs such as ocrelizumab, veltuzumab, ocaratuzumab, ublituximab, or PRO131921 with rituximab were identified. The concomitant chemotherapy regimens included CHOP (cyclophosphamide, doxorubicin, vincristine, and prednisone), ACVBP (doxorubicin, cyclophosphamide, vindesine, bleomycin, and prednisone), CVP (cyclophosphamide, vincristine, and prednisone), Bendamustine, DHAP (cisplatin, cytarabine, dexamethasone), and BEAM (carmustine, etoposide, cytarabine, and melphalan). Concomitant chemotherapy regimens were completely same between the two arms in each trial. A total of 5261 B-NHL patients with a median age of 48–62 years were included in the analysis. Seven trials included only relapsed/refractory patients, and other four trials included only previously untreated patients. Patients demographic and baseline disease characteristics were well balanced between arms in all included trails. Detailed characteristics of the included 11 trials are summarized in Table 1. The results of quality assessment are shown in Supplementary Table S1.

**Table 1:** Characteristics of the 11 RCTs included in meta-analyses.

Trial	Enroll period	Study design	Eligible patients	Median follow-up (months)	Comparability between arms	Intervention arms	Dose	Concomitant chemotherapy regimens	Patients randomized/analyzed	Median age (range, years)	Gender (male%)
GAINED trial^18^ (NCT01659099)	Sep 2012–Jul 2015	Open-label, multicenter, phase III	Previously untreated CD20^+^ DLBCL	25.2	NS^a^ (except a higher proportion of male patients in the obinutuzumab arm, p < 0.016)	Obinutuzumab	1000 mg iv on day 1 and 8 of cycles 1–2, and on day 1 of cycles 3–4	ACVBP/CHOP	336/336	48 (18–60)	60.4
						Rituximab	375 mg/m^2^ iv on day 1 for 4 cycles	ACVBP/CHOP	334/334	48 (18–60)	50.9
GALLIUM study^19–21^ (NCT01332968)	Jul 2011–Feb 2014	Open-label, multicenter, phase III	Previously untreated CD20^+^ FL	57.3	NS^a^	Obinutuzumab	1000 mg iv on days 1, 8 and 15 of cycle 1, and on day 1 of cycles 2–6/8	CHOP/CVP/Bendamustine	601/601	60 (26–88)	47.1
						Rituximab	375 mg/m^2^ iv on day 1 for 6/8 cycles	CHOP/CVP/Bendamustine	601/601	58 (23–85)	46.6
GAUSS study^22^ (NCT00576758)	Jul 2009–Aug 2010	Open-label, multicenter, phase II	Relapsed CD20^+^ indolent B-cell NHL (FL, MZL, LPL, SLL)	32	NS^a^	Obinutuzumab	1000 mg iv once per week for 4 weeks	None	88/88	62 (33–84)	50
						Rituximab	375 mg/m^2^ iv once per week for 4 weeks	None	87/87	60 (38–80)	52
GOYA study^23^ (NCT01287741)	Jul 2011–Jun 2014	Open-label, multicenter, phase III	Previously untreated CD20^+^ DLBCL	29	NS^a^	Obinutuzumab	1000 mg iv on days 1, 8 and 15 of cycle 1, and on day 1 of cycles 2–6/8	CHOP	706/706	62 (18–86)	52.3
						Rituximab	375 mg/m^2^ iv on day 1 for 6/8 cycles	CHOP	712/712	62 (18–83)	53.8
HOMER study^24^	NA^b^	Open-label, multicenter, phase III	Relapsed CD20^+^ indolent B-Cell NHL (98% patients had FL)	NA^b^	NS^a^	Ofatumumab	1000 mg iv once per week for 4 weeks	None	205/205	NA^b^	NA^b^
						Rituximab	375 mg/m^2^ iv once per week for 4 weeks	None	204/204	NA^b^	NA^b^
ORCHARRD study^25^ (NCT01014208)	Mar 2010–Dec 2013	Open-label, multicenter, phase III	Relapsed/Refractory CD20^+^ DLBCL	10.9	NS^a^	Ofatumumab	1000 mg iv on day1 and 8 of cycle 1, and on day1 of cycle 2–3	DHAP	222/222	58 (23–83)	62
						Rituximab	375 mg/m^2^ iv on day1 and 8 of cycle 1, and on day1 of cycle 2–3	DHAP	225/223	56 (18–79)	61
BMT CTN 0401 trial^26^ (NCT00329030)	Jan 2006–Jul 2009	Open-label, multicenter, phase III	Relapsed/Refractory CD20^+^ DLBCL	25.5	NS^a^	^131^I-Tositumomab	Dosimetric dose: 5 mCi; therapeutic dose: 0.75 Gy	BEAM	111/111	57 (20–75)	61.3
						Rituximab	375 mg/m^2^ iv for 2 doses	BEAM	113/113	59 (24–77)	65.5
Quackenbush 2015^27^ (NCT00268983)	Aug 2004–Aug 2006	Open-label, multicenter, phase III	Relapsed CD20^+^ FL	62.0–91.5	NS^a^	^131^I-Tositumomab	Dosimetric dose: 450 mg of unlabeled TST, 35 mg of TST labeled with 5 mCi of ^131^I; therapeutic dose: 450 mg of unlabeled TST, 35 mg of TST labeled with patient-specific activity of ^131^I to deliver 0.65/0.75 Gy whole-body dose	None	9/8	54 (42–64)	NA^b^
						Rituximab	375 mg/m^2^ iv once per week for 4 weeks	None	6/6	61 (41–78)	NA^b^
SWOG S0016 study^28–29^ (NCT00006721)	Mar 2001–Sep 2008	Open-label, multicenter, phase III	Previously untreated CD20^+^ FL	123.6	NS^a^	^131^I-Tositumomab	Dosimetric dose: 450 mg of unlabeled TST, 35 mg of TST labeled with 5 mCi of ^131^I; therapeutic dose: 450 mg of unlabeled TST, 35 mg of TST labeled with patient-specific activity of ^131^I to deliver 0.65/0.75 Gy whole-body dose	CHOP	264/264	53.4	56
						Rituximab	375 mg/m^2^ iv for 6 doses	CHOP	267/267	54.5	53
Khouri 2015^30^	2007–2010	Randomized trial	Relapsed CD20^+^ DLBCL	50.4–58.8	NS^a^	^90^Y-Ibritumomab tiuxetan	0.4 mCi/Kg	BEAM	14/14	NA^b^	NA^b^
						Rituximab	1,000 mg/m^2^ iv for 2 doses	BEAM	16/16	NA^b^	NA^b^
Witzig 2002^31–32^	NA^b^	Multicenter, phase III	Relapsed/Refractory CD20^+^ low grade or follicular or transformed NHL	44	NS^a^	^90^Y-Ibritumomab tiuxetan	Dosimetric dose: 1.6 mg of ibritumomab tiuxetan labeled with 5 mCi of ^111^In iv; therapeutic dose: ^90^Y-ibritumomab tiuxetan 0.4 mCi/kg iv	None	73	60 (29–80)	48
						Rituximab	375 mg/m^2^ iv once per week for 4 weeks	None	70	57 (36–78)	50

*DLBCL* diffuse large B-cell lymphoma, *ACVBP* doxorubicin, cyclophosphamide, vindesine, bleomycin, and prednisone, *CHOP* cyclophosphamide, doxorubicin, vincristine, and prednisone, *FL* follicular lymphoma, *CVP* cyclophosphamide, vincristine, and prednisone, *NHL* non-Hodgkin lymphoma, *MZL* marginal zone lymphoma, *LPL* lymphoplasmacytic lymphoma, *SLL* small lymphocytic lymphoma, *DHAP* cisplatin, cytarabine, dexamethasone, *BEAM* carmustine, etoposide, cytarabine, and melphalan, ^*131*^*I* iodine-131, *TST* Tositumomab, ^*90*^*Y* yttrium-90, ^*111*^*In* indium-111.

^a^No significant differences.

^b^Not available.

### Obinutuzumab versus Rituximab

**Figure 2:**
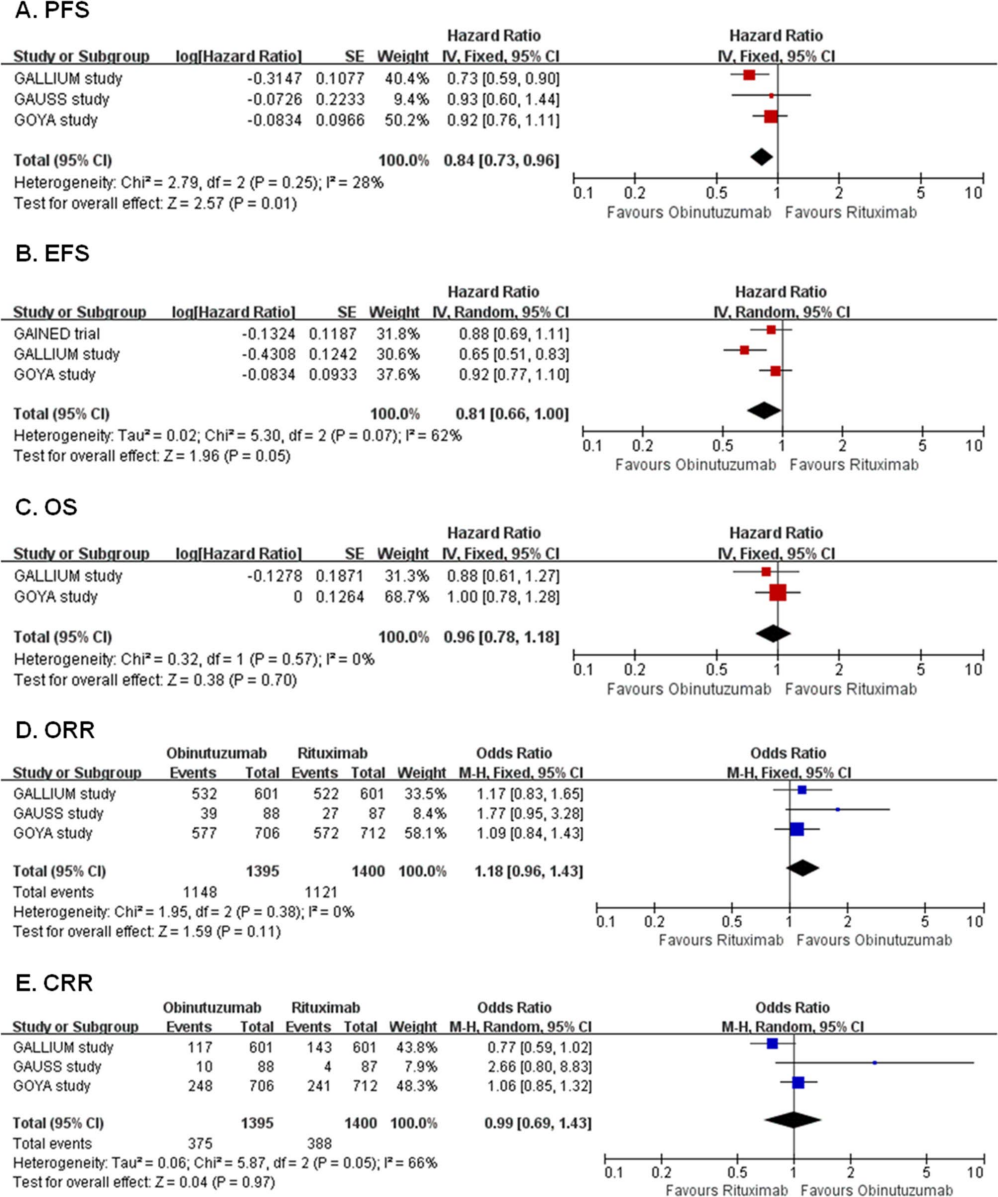
Forest plots of obinutuzumab versus rituximab. Forest plot of the meta-analysis that estimating the ORs and HRs with their corresponding 95% CIs for the obinutuzumab group, compared with that in the rituximab group. *PFS* progression-free survival, *EFS* event-free survival, *OS* overall survival, *ORR* overall response rate, *CRR* complete response rate, *HRs* hazard ratios, *ORs* odds ratios, *95% CI* 95% confidence interval.

Four RCTs with 3465 patients compared obinutuzumab with rituximab in induction therapy of CD20^+^ B-NHL. Meta-analyses showed that compared with rituximab, obinutuzumab significantly prolonged PFS (HR 0.84, 95% CI 0.73–0.96, *P* = 0.01, Fig. 2A). There were no significant differences between arms in EFS (HR 0.81, 95% CI 0.66–1.00, *P* = 0.05), OS (HR 0.96, 95% CI 0.78–1.18, *P* = 0.70), ORR (OR 1.18, 95% CI 0.96–1.43, *P* = 0.11) and CRR (OR 0.99, 95% CI 0.69–1.43, *P* = 0.97) (Fig. 2B–E). The results of all included efficacy outcomes for this comparison are summarized in Table 2.

**Table 2:** Summary of results of all included efficacy outcomes for each comparison.

Outcome	Comparison	Overall effects	
**PFS**		**HR (95% CI)**	***P***
	Obinutuzumab versus Rituximab	0.84 (0.73–0.96)	0.01*
	Ofatumumab versus Rituximab	1.13 (0.95–1.34)	0.17
	^131^I-tositumomab versus Rituximab	0.80 (0.53–1.21)	0.29
	^90^Y-ibritumomab tiuxetan versus Rituximab	0.79 (0.54–1.18)	0.25
**EFS**		**HR (95% CI)**	***P***
	Obinutuzumab versus Rituximab	0.81 (0.66–1.00)	0.05
	Ofatumumab versus Rituximab	1.10 (0.89–1.35)	0.37
**DFS**		**HR (95% CI)**	***P***
	^90^Y-ibritumomab tiuxetan versus Rituximab	1.32 (0.30–5.87)	0.71
**OS**		**HR (95% CI)**	***P***
	Obinutuzumab versus Rituximab	0.96 (0.78–1.18)	0.70
	Ofatumumab versus Rituximab	0.93 (0.74–1.17)	0.54
	^131^I-tositumomab versus Rituximab	1.04 (0.56–1.90)	0.91
	^90^Y-ibritumomab tiuxetan versus Rituximab	1.31 (0.51–3.36)	0.57
**ORR**		**OR (95% CI)**	***P***
	Obinutuzumab versus Rituximab	1.18 (0.96–1.43)	0.11
	Ofatumumab versus Rituximab	0.73 (0.55–0.96)	0.02*
	^131^I-tositumomab versus Rituximab	1.25 (0.40–3.93)	0.70
	^90^Y-ibritumomab tiuxetan versus Rituximab	3.07 (1.47–6.43)	0.003*
**CRR**		**OR (95% CI)**	***P***
	Obinutuzumab versus Rituximab	0.99 (0.69–1.43)	0.97
	Ofatumumab versus Rituximab	0.66 (0.41–1.07)	0.09
	^131^I-tositumomab versus Rituximab	2.93 (0.27–32.18)	0.38
	^90^Y-ibritumomab tiuxetan versus Rituximab	2.08 (0.97–4.45)	0.06

*PFS* progression-free survival, *EFS* event-free survival, *OS* overall survival, *ORR* overall response rate, *CRR* complete response rate, *HR* hazard ratio, *OR* odds ratios, *95% CI* 95% confidence interval.

*Statistically significant results (*P* < 0.05).

For the incidences of AEs, meta-analyses showed that obinutuzumab arm had higher incidences of total AEs, grade 3–5 AEs, serious AEs, fatal AEs, total infusion-related reaction (IRR), grade 3–5 IRR, all grades of neutropenia, grade 3–5 neutropenia, grade 3–5 thrombocytopenia, pyrexia, diarrhea, headache, grade 3–5 infections, chills, and insomnia. The incidences of other AEs were comparable between the two arms (Supplementary Table S2).

Subgroup analysis showed that obinutuzumab seems significantly improved PFS especially in patients with previously untreated disease, follicular lymphoma (FL) subtype, female gender, white race, bulky disease, late stage, better Eastern Cooperative Oncology Group performance status (ECOG PS), intermediate international prognostic index (IPI) and when used in combination with bendamustine, However, the subgroup differences were not statistically significant (Supplementary Table S3). Regarding OS, ORR, and CRR, subgroup analyses showed clearly no significant interaction between the effect of obinutuzumab versus rituximab with treatment history, disease subtype and concomitant chemotherapy regimen (Supplementary Table S3).

### Ofatumumab versus Rituximab

**Figure 3:**
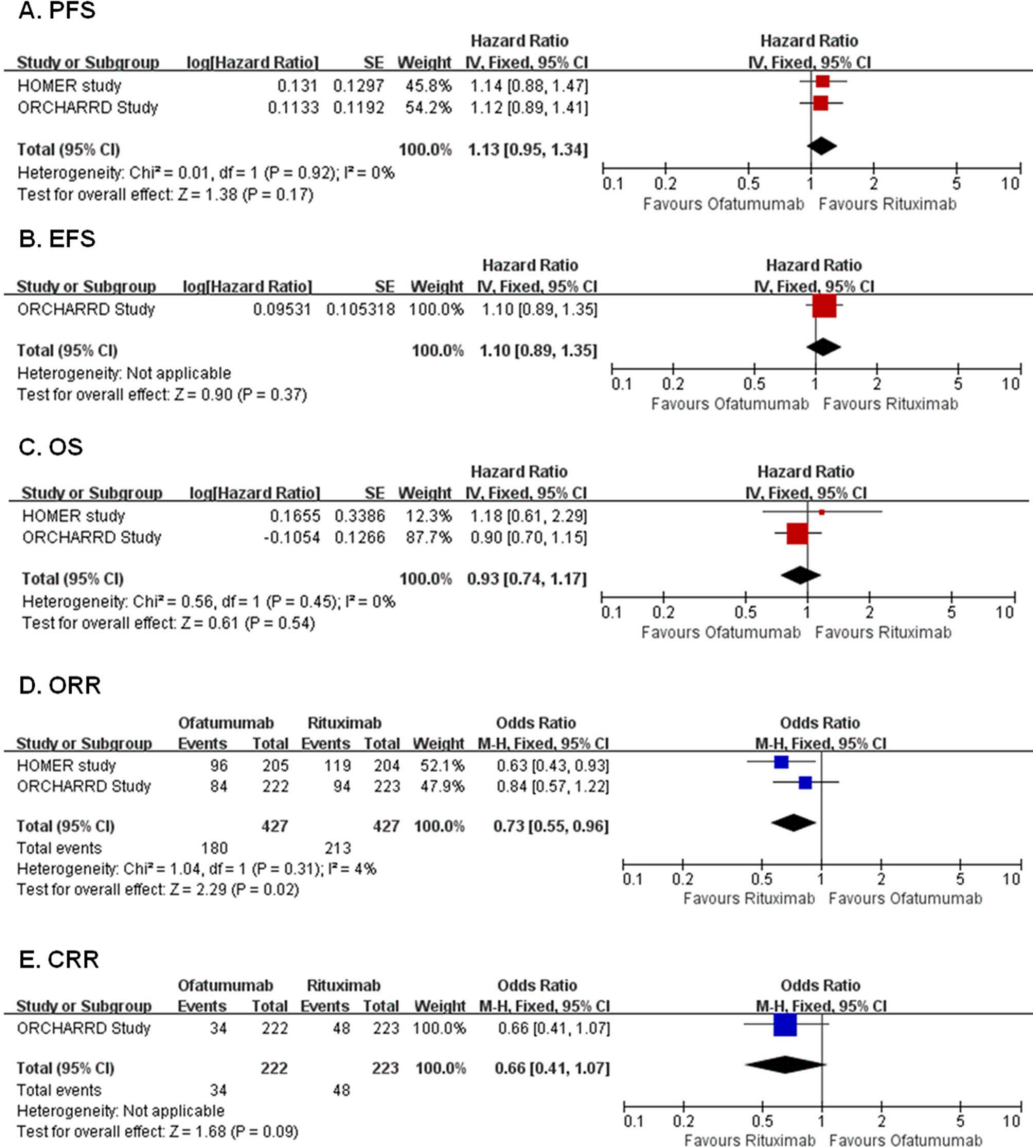
Forest plots of ofatumumab versus rituximab. Forest plot of the meta-analysis that estimating the ORs and HRs with their corresponding 95% CIs for the ofatumumab group, compared with that in the rituximab group. *PFS* progression-free survival, *EFS* event-free survival, *OS* overall survival, *ORR* overall response rate, *CRR* complete response rate, *HRs* hazard ratios, *ORs* odds ratios, *95% CI* 95% confidence interval.

Two RCTs with 854 patients compared ofatumumab with rituximab in induction therapy of relapsed/refractory CD20^+^ B-NHL. There were no significant differences between arms regarding to PFS (HR 1.13, 95% CI 0.95–1.34, *P* = 0.17), EFS (HR 1.10, 95% CI 0.89–1.35, *P* = 0.37), OS (HR 0.93, 95% CI 0.74–1.17, *P* = 0.54) and CRR (OR 0.66, 95% CI 0.41–1.07, P = 0.09) (Fig. 3A–C,E). As for ORR, ofatumumab was inferior to rituximab (OR 0.73, 95% CI 0.55–0.96, P = 0.02, Fig. 3D). The results of all included efficacy outcomes for this comparison are summarized in Table 2.

For the incidences of AEs, ofatumumab was associated with higher incidences of infusion-related AEs, grade 3–5 infusion-related AEs, gastrointestinal disorders, AEs leading to dose interruptions and rash, but lower incidences of pyrexia. The incidences of other AEs were comparable between arms (Supplementary Table S4).

Since the included two trials only enrolled relapsed/refractory patients, so subgroup analyses for PFS, OS, ORR and CRR were only performed according disease subtype and concomitant chemotherapy regimen. The results showed there were no significant interaction between the effect of ofatumumab versus rituximab with disease subtype and concomitant chemotherapy regimen (Supplementary Table S5).

### ^131^I-tositumomab versus Rituximab

**Figure 4:**
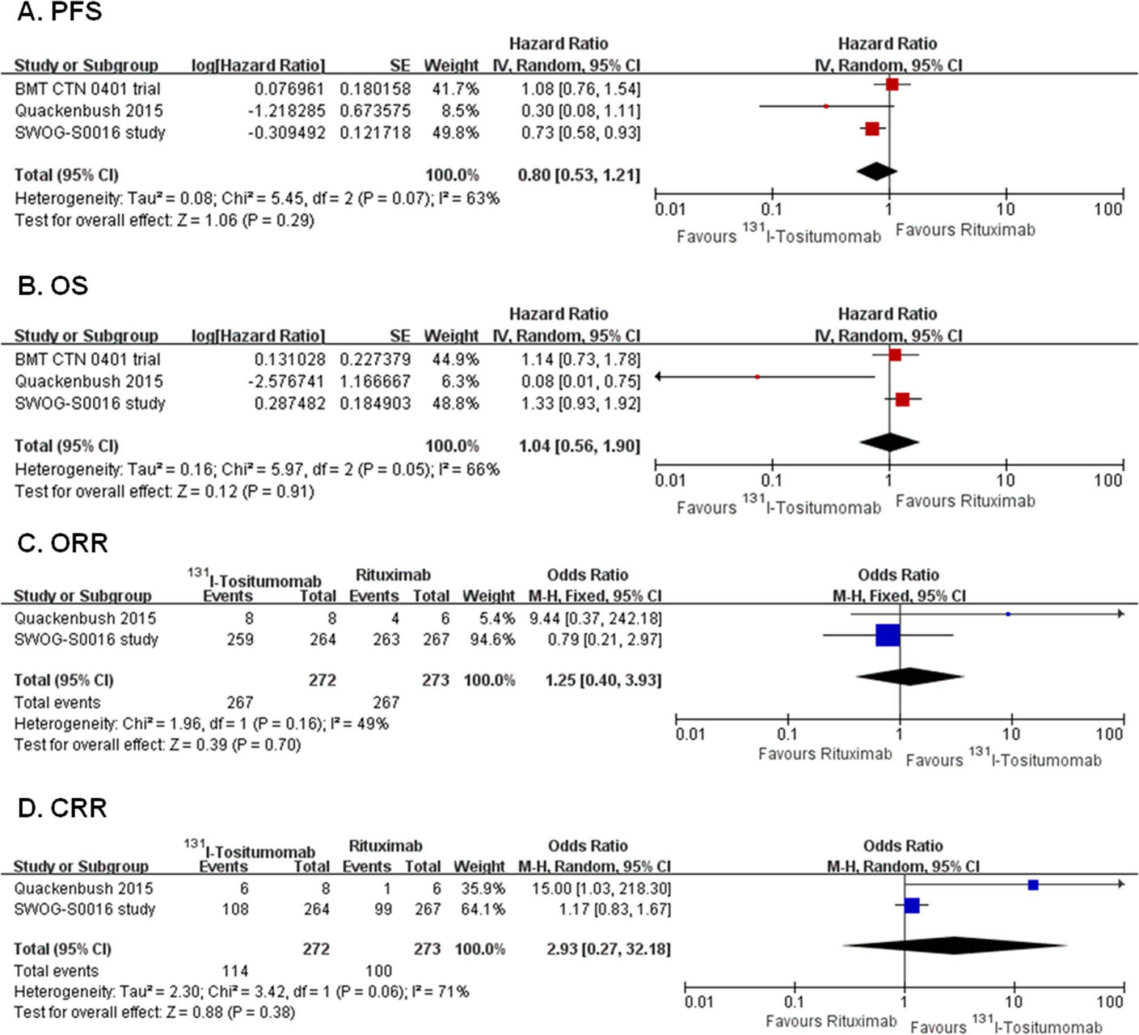
Forest plots of ^131^I-tositumomab versus rituximab. Forest plot of the meta-analysis that estimating the ORs and HRs with their corresponding 95% CIs for the ^131^I-tositumomab group, compared with that in the rituximab group. *PFS* progression-free survival, *OS* overall survival, *ORR* overall response rate, *CRR* complete response rate, *HRs* hazard ratios, *ORs* odds ratios, *95% CI* 95% confidence interval.

Three RCTs with 769 patients compared the efficacy and safety of ^131^I-tositumomab with rituximab in induction therapy of B-NHL. Meta-analyses showed that there were no significant differences between arms in PFS (HR 0.80, 95% CI 0.53–1.21, *P* = 0.29,), OS (HR 1.04, 95% CI 0.56–1.90, *P* = 0.91), ORR (OR 1.25, 95% CI 0.40–3.93, *P* = 0.70) and CRR (OR 2.93, 95% CI 0.27–32.18, *P* = 0.38) (Fig. 4A–D). The results of all included efficacy outcomes for this comparison are summarized in Table 2.

For the incidences of AEs, ^131^I-tositumomab arm was associated with higher incidences of total grade 3–5 AEs, grade 3–5 thrombocytopenia, grade 3–5 febrile neutropenia, and grade 3–5 mucositis. Other AEs were comparable between these two arms (Supplementary Table S6).

Subgroup analyses for PFS, OS, ORR and CRR showed no significant interaction between the effect of ^131^I-tositumomab versus rituximab with treatment history, disease subtype and concomitant chemotherapy regimen (Supplementary Table S7).

Since the trial conducted by Quackenbush et al*.* enrolled only 14 patients and was terminated due to lack of feasibility, sensitivity analysis was performed by omitting this trial. The results of sensitivity analyses showed that the pooled HRs were 0.87 (95% CI 0.60–1.27, *P* = 0.47) for PFS and 1.25 (95% CI 0.95–1.66, *P* = 0.12) for OS, the pooled ORs were 0.79 (95% CI 0.21–2.97, *P* = 0.72) for ORR and 1.17 (95% CI 0.83–1.67, *P* = 0.37) for CRR, which were all similar with the main analyses.

### ^90^Y-ibritumomab tiuxetan versus Rituximab

**Figure 5:**
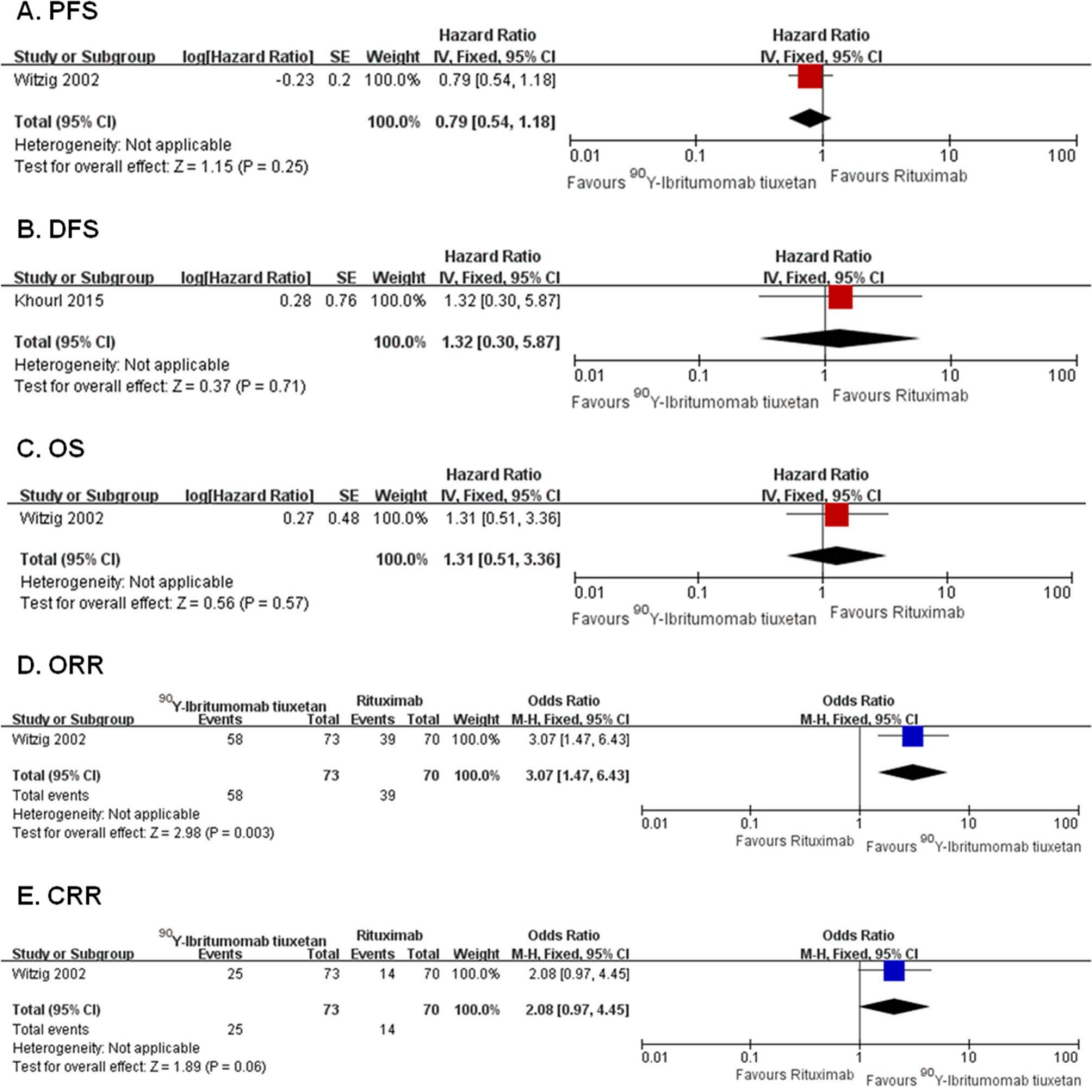
Forest plots of ^90^Y-ibritumomab tiuxetan versus rituximab. Forest plot of the meta-analysis that estimating the ORs and HRs with their corresponding 95% CIs for the ^131^I-tositumomab group, compared with that in the rituximab group. *PFS* progression-free survival, *DFS* disease-free survival, *OS* overall survival, *ORR* overall response rate, *CRR* complete response rate, *HRs* hazard ratios, *ORs* odds ratios, *95% CI* 95% confidence interval.

Two RCTs with 173 patients compared ^90^Y-ibritumomab tiuxetan with rituximab in induction therapy of B-NHL. Survival outcomes including PFS (HR 0.79, 95% CI 0.54–1.18, *P* = 0.25), OS (HR 1.31, 95% CI 0.51–3.36, *P* = 0.57), and DFS (HR 1.32, 95% CI 0.30–5.87, *P* = 0.71) were comparable between ibritumomab arm and rituximab arm (Fig. 5A–C). A higher ORR of ^90^Y-ibritumomab tiuxetan compared with rituximab was noted, with an estimate OR of 3.07 (95% CI 1.47–6.43, *P* = 0.003) (Fig. 5D). The CRR was comparable between arms, with an estimate OR of 2.08 (95% CI 0.97–4.45, *P* = 0.06) (Fig. 5E). The results of all included efficacy outcomes for this comparison are summarized in Table 2.

There were no significant differences between two arms regarding the incidences of non-hematologic AEs excepted for higher incidences of nausea and vomiting in the ^90^Y-ibritumomab tiuxetan arm (Supplementary Table S8).

## Discussion

Since its initial approval in 1997, rituximab has revolutionized the treatment of CD20^+^ B-NHL. Over the last two decades, new anti-CD20 mAbs are emerging that are expected to have improved biological advantages and may be more effective compared with rituximab. This meta-analysis of 11 RCTs including 5261 CD20^+^ B-NHL patients compared the efficacy and safety of new anti-CD20 mAbs including obinutuzumab, ofatumumab, ^131^I-tositumomab and ^90^Y-ibritumomab with rituximab in induction therapy. The results demonstrated that compared with rituximab, obinutuzumab had a significant improvement in PFS although increased the incidences of various AEs, and ^90^Y-ibritumomab achieved a higher ORR without improvement of PFS. Subgroup analyses showed that the superiority of obinutuzumab over rituximab in PFS seemed to be more significant in previously untreated patients, FL subtype, female patients, white patients, late-stage patients and when used in combination with bendamustine. The two other agents, ofatumumab and ^131^I-tositumomab, did not show any improvement in all analyzed efficacy outcomes including PFS, OS, ORR and CRR, but had higher incidences of various AEs. Our results suggested that in induction therapy of patients with previously untreated late-stage FL, obinutuzumab may be a preferred choice since it can significantly prolong PFS compared with rituximab. However, the increment in the risk of AEs and cost should be taken into consideration when make decisions in clinical practice^33,34^.

The predominant mechanisms of how anti-CD20 mAbs kill target cells include complement-dependent cytotoxicity (CDC), antibody-dependent cellular cytotoxicity (ADCC), and direct cell death (DCD)^11,35^. According to their ability to induce the clustering of CD20 into lipid rafts, the anti-CD20 mAbs can be largely classified into two groups, type I and type II^8^. After binging to target cells with the Fc portions, type I anti-CD20 mAbs (such as rituximab and ofatumumab) induce the redistribution of CD20 into lipid rafts on cell membrane, leading to stronger C1q binding and more effective activation of complement cascade, initiating prominent CDC^8,11,36^. However, this binding mode trigger minimal DCD^8^. On the contrary, type II anti-CD20 mAbs do not induce the redistribution of CD20 into lipid rafts and do not evoke significant CDC, but they induce more potent DCD^10–12^. ADCC occurs after the interaction of the Fc portions from antibodies with the FcR expressed on effector cells (such as neutrophils, natural killer cells and macrophages)^11^. Modifications on the Fc portions of new anti-CD20 mAbs result in greater ADCC through enhancing its binding affinity to FcR^10^. Collectively, anti-CD20 mAbs exert antitumor effects through different mechanisms due to their structural variations. Compared with rituximab, modifications of new anti-CD20 mAbs significantly improved efficacy in preclinical studies.

Obinutuzumab is a type II anti-CD20 mAb that does not cause the clustering of CD20 into lipid rafts on the membrane and shows more potent direct cell death (DCD) and antibody-dependent cellular cytotoxicity (ADCC)^8^. Besides, the glycoengineering process caused defucosylation in the Fc region of obinutuzumab, which can enhance binding affinity to the FcR on immune effector cells and provoked more effective ADCC^11^. This may explain why obinutuzumab improve PFS of CD20^+^ NHL patients compared with rituximab. Although obinutuzumab-based therapy significantly prolonged PFS compared with rituximab-based therapy, no difference in OS was observed between the obinutuzumab arm and the rituximab arm. A possible reason is that the data of OS are still immature. The 4-year OS rates of patients with FL were as high as 92.6% in obinutuzumab arm and 90.3% in rituximab arm, the median OS had not been reached^21^. Prolonged follow-up is required to verify that if obinutuzumab-based therapy could benefit in OS compared with rituximab. Another possible explanation is that obinutuzumab was associated higher risk of fatal AEs compared with rituximab^20,22,23^.

Ofatumumab is a type I anti-CD20 mAb that binds to a unique epitope of CD20 and shows greater binding avidity than rituximab^9,37^. Preclinical studies have demonstrated that ofatumumab is more effective than rituximab in killing target cells regardless of FcR polymorphisms and the levels of CD20 expression^38^. But our meta-analysis failed to demonstrate any superiority of ofatumumab over rituximab in patients with relapsed CD20^+^ B-NHL. The lack of improvement in clinical outcomes with ofatumumab-based therapy in patients with relapsed CD20^+^ B-NHL may result from the resistance to anti-CD20 mAbs since only relapsed patients were included in the meta-analysis. Clinical trials aiming to compare the efficacy and safety of ofatumumab with rituximab in patients with previously untreated CD20^+^ B-NHL are required.

Novel agents that conjugate radioisotope to anti-CD20 mAbs, such as ^131^I-tositumomab (Bexxar) and ^90^Y-ibritumomab tiuxetan (Zevalin), also have been developed and approved for treatments of B-NHL. These agents could cause a crossfire effect and eradicate nearby tumor cells that are not targeted by antibody but are affected by radiation^14^. Radioimmunotherapy with ^131^I-tositumomab could achieve a high response rate of 47–68% in heavily pretreated NHL patients^39^. However, the results of our meta-analysis did not demonstrate any significant differences in PFS, OS and response rate between the ^131^I-tositumomab arm and the rituximab arm due to the lack of benefits for ^131^I-tositumomab-based therapy in patients with relapsed DLBCL. In addition, the manufacture of tositumomab was discontinued for commercial reason in 2014 and it is unavailable now^40^. Another radio-labeled anti-CD20 mAb ^90^Y-ibritumomab tiuxetan was compared with rituximab in the therapy of relapsed B-NHL and this trial was included in our systematic review. Results showed that ^90^Y-ibritumomab achieved higher ORR, similar PFS, OS and CRR. According to a multicenter phase III randomized trial, consolidation therapy with ^90^Y-ibritumomab tiuxetan in patients with FL in first remission notably prolonged median PFS compared with no further treatment. Another randomized trial has compared consolidation therapy with ^90^Y-ibritumomab tiuxetan versus rituximab in FL patients that achieved CR or partial response (PR) after R-CHOP treatment. These results indicated that ^90^Y-ibritumomab tiuxetan was inferior to rituximab in PFS^41^. Therefore, although ^90^Y-ibritumomab tiuxetan was associated with slightly higher response rate as induction therapy for relapsed patients but with inferior PFS as consolidation therapy for patients in remission, the benefits of ^90^Y-ibritumomab tiuxetan over rituximab are still conflicting due to lack of enough evidence.

AE is another aspect that need to be considered for evaluating a new drug. In our meta-analysis, all the new CD20 mAbs showed higher incidences of AEs compared with rituximab. Overall, these biologics appear to be well tolerated and lead to fewer AEs compared with other more conventional therapies and chemotherapeutic regimens^42^. But serious AEs associated with the use of rituximab have already been an important consideration during clinical practice^43^. What’s more, the increasingly widespread and potentially prolonged use of rituximab and new CD20 mAbs poses a new challenge. Therefore, knowledge of serious AEs related to new CD20 mAbs antibodies is essential. Our results provide a clue for the awareness of AEs of new CD20 mAbs during their propagation.

To the best of our knowledge, this is the first systematic review and meta-analysis that integrated currently all available published data to compare the efficacy and safety of new anti-CD20 mAbs with rituximab. Totally 5261 CD20^+^ B-NHL participants with diverse racial and ethnic groups were included. Most of the included trials are with high quality and enough time of follow-up. What is more, in consideration of potential confounders such as treatment history, subtype, gender and concomitant chemotherapy, subgroup analysis was conducted to investigate the internal validity of this meta-analysis, indicating the consistency between different subgroups. All these characteristics enabled us to get a reliable result.

Nevertheless, there are some limitations in this study. Firstly, data were pooled from participants with different disease subtypes and disease status. For the limit number of currently available clinical trials, we have to enrolled patients with any types of CD20^+^ B-NHL including newly diagnosed or relapsed FL, DLBCL, transformed NHL, marginal zone lymphoma (MZL) and other indolent B-NHLs. Although they have lots of in common and no statistically significant subgroup differences are observed, the results need to be verified in more clinical trials focusing on a certain subtype of B-NHL. In addition, the absence of individual patient data did not allow further analysis of the benefits for survival outcomes in different subgroups based on disease subtypes, disease status, risk stratification and molecular characteristics. Another potential limitation of this study is that we pooled results from trials of different phases. The inclusion of earlier phase studies would increase the risk of investigator bias and confound the pooled analysis. Actually, most of the studies we included (9/11, 82%) are phase III trials except for a phase II trial (the GAUSS study) and a trial that did not provide information about study phase (Khouri 2015). The risk of investigator bias of the GAUSS study is low since it incorporated a blinded response assessment by an independent review facility (IRF) to better guide phase III trial planning^22^. Therefore, we think confounding resulted from the inclusion of earlier phase studies in our analysis is relatively small.

## Conclusions

In conclusion, we have compared new CD20 mAbs with rituximab based on all current available published data by systematic review and meta-analysis. Obinutuzumab was demonstrated with a significant improvement in PFS, but no improvements in OS, ORR and CRR, and an increment in the incidences of AEs. Ofatumumab showed comparable results in PFS, OS and CRR, but a lower ORR and higher incidences of AEs. ^131^I-tositumomab yielded similar results with rituximab regarding PFS, OS, ORR and CRR but was associated with higher incidences of AEs. ^90^Y-ibritumomab achieved a higher ORR, similar PFS, OS and CRR, but was associated with higher incidences of AEs. These results might facilitate clinical decision making and assist in the design and interpretation of future trials.

## Supplementary Information

Supplementary Information.

## Data Availability

All data were extracted from already published articles and their Supplementary files, which can be accessed by everyone.
