# Supplementary material for: Efficacy and safety of new anti-CD20 monoclonal antibodies versus rituximab for induction therapy of CD20+ B-cell non-Hodgkin lymphomas: a systematic review and meta-analysis
Source: Sci Rep. 2021 Feb 5;11:3255. doi: 10.1038/s41598-021-82841-w (PMC7864901; doi:10.1038/s41598-021-82841-w)
Supplement: Supplementary file 1 — Supplementary Information. [file 41598_2021_82841_MOESM1_ESM.pdf]

Efficacy and safety of new anti-CD20 monoclonal antibodies versus rituximab for induction therapy of CD20$^+$ B-cell non-Hodgkin lymphomas: a systematic review and meta-analysis

Chengxin Luo[1,2], Guixian Wu[1,2], Xiangtao Huang[1,2], Yanni Ma[1,2], Yali Zhang[1,2], Qiuyue Song[3], Mingling Xie[1,2], Yanni Sun[1,2], Yarui Huang[1,2], Zhen Huang[1,2], Yu Hou[1,2], Shuangnian Xu[1,2*], Jieping Chen[1,2*], Xi Li [4*].

[1]Center for Hematology, Southwest Hospital, Third Military Medical University, Chongqing, China; [2] Key Laboratory of Cancer Immunotherapy of Chongqing, Chongqing, China; [3]Department of Health Statistics, Third Military Medical University, Chongqing, China. [4]Institute of Infectious Disease, Southwest Hospital, Third Military Medical University, Chongqing, China.

[*]Correspondence to: Prof. Shuangnian Xu, Prof. Jieping Chen, and Prof. Xi Li, Center for Hematology, Southwest Hospital, Third Military Medical University, #30 Gaotanyan Street, Shapingba District, Chongqing, 400038, China; Prof. Xi Li, Institute of Infectious Disease, Southwest Hospital, Third Military Medical University, #30 Gaotanyan Street, Shapingba District, Chongqing, 400038, China.

Tel: +86-15223038417

E-mail: xushuangnian@tmmu.edu.cn (Shuangnian Xu); chenjpxn@163.com (Jieping Chen); lixi1988xn@163.com (Xi Li)

Appendix. Search Strategies for MEDLINE, Embase, and Cochrane Library.

MEDLINE:

1	exp Lymphoma/
2	(lymphom$ or linfom$).tw,kf,ot.
3	exp Hematologic Neoplasms/
4	(lympho$ adj2 (neoplasm$ or malign$ or tumor$ or tumour$ or sarcom$)).tw,kf,ot.
5	(lympha$ adj2 (neoplasm$ or malign$ or tumor$ or tumour$ or sarcom$)).tw,kf,ot.
6	(hemato$ adj (malign$ or neoplas$)).tw,kf,ot.
7	(haemato$ adj (malign$ or neoplas$)).tw,kf,ot.
8	exp Lymphoma, Non-Hodgkin/
9	(nonhodgkin$ or non-hodgkin$ or non hodgkin$ or no hodgkin$ or nhl$).tw,kf,ot.
10	mycos$ fungo$.tw,kf,ot.
11	sezary$.tw,kf,ot.
12	granulom$.tw,kf,ot.
13	burkitt$.tw,kf,ot.
14	lymphosarcom$.tw,kf,ot.
15	reticulosarcom$.tw,kf,ot.
16	reticulum-cell sarcom$.tw,kf,ot.
17	exp Disease, Hodgkin/
18	hodgkin$.tw,kf,ot.
19	Germinoblastom$.tw,kf,ot.
20	Reticulolymphosarcom$.tw,kf,ot.
21	(malignan$ adj2 (lymphogranulom$ or granulom$)).tw,kf,ot.
22	or/1-21
23	rituximab$.tw,kf,ot,nm.
24	mabt$.tw,kf,ot,nm.
25	ritux$.tw,kf,ot,nm.
26	idec$.tw,kf,ot,nm.
27	(idec-c2b8 or idecc2b8).tw,kf,ot,nm.
28	gp2013.tw,kf,ot,nm.
29	ofatumumab$.tw,kf,ot,nm.
30	arzerr$.tw,kf,ot.
31	(humax-cd20 or humaxcd20).tw,kf,ot,nm.
32	ocrelizumab$.tw,kf,ot,nm.
33	(r-1594 or r1594).tw,kf,ot,nm.
34	pr070769.tw,kf,ot,nm.
35	veltuzumab$.tw,kf,ot,nm.
36	(immu-106 or immu106).tw,kf,ot,nm.

37	obinutuzumab$.tw,kf,ot,nm.
38	gazyva$.tw,kf,ot,nm.
39	(r-7159 or r7159).tw,kf,ot,nm.
40	(ro-5072759 or ro5072759).tw,kf,ot,nm.
41	(ga-101 or ga101).tw,kf,ot,nm.
42	ocaratuzumab$.tw,kf,ot,nm.
43	ame133.tw,kf,ot,nm.
44	(ly2469298 or ly2469298).tw,kf,ot,nm.
45	ublituximab$.tw,kf,ot,nm.
46	(tg-1101 or tg1101).tw,kf,ot,nm.
47	pro131921.tw,kf,ot,nm.
48	tositumomab$.tw,kf,ot,nm.
49	bexxar$.tw,kf,ot,nm.
50	(iodine-131 or I-131 or 131I).tw,kf,ot,nm.
51	(anti-B1 or anti-CD20).tw,kf,ot,nm.
52	50 and 51
53	ibritumomab$.tw,kf,ot,nm.
54	zevalin$. tw,kf,ot,nm.
55	(idec-y2b8 or idecy2b8).tw,kf,ot,nm.
56	exp Antigens, CD20/
57	((cd20 or cd-20 or cd 20) adj3 antibod$).tw,kf,ot,nm.
58	(anti-cd20 or anti cd20).tw,kf,ot,nm.
59	(anticd20 or anti-cd-20 or anticd-20).tw,kf,ot,nm.
60	or/23-49 52-59
61	randomized controlled trial.pt.
62	controlled clinical trial.pt.
63	randomized.ab.
64	placebo.ab.
65	drug therapy.fs.
66	randomly.ab.
67	trial.ab.
68	groups.ab.
69	or/61-68
70	humans.sh.
71	69 and 70
72	22 and 60 and 71

Embase:

1	'lymphoma'/exp
2	(lymphom* OR linfom*):ab,ti
3	'hematologic neoplasms'/exp
4	(lympho* NEAR/2 (neoplasm* OR malign* OR tumor* OR tumour* OR sarcom*)):ab,ti
5	(lympha* NEAR/2 (neoplasm* OR malign* OR tumor* OR tumour* OR sarcom*)):ab,ti
6	(hemato* NEAR/1 (malign* OR neoplas*)):ab,ti
7	(haemato* NEAR/1 (malign* OR neoplas*)):ab,ti
8	'non-hodgkin lymphoma'/exp
9	(nonhodgkin* OR non-hodgkin* OR ‘non hodgkin*’ OR hodgkin* OR nhl*):ab,ti
10	‘mycos* fungo*’:ab,ti
11	sezary*:ab,ti
12	granulom*:ab,ti
13	burkitt*:ab,ti
14	lymphosarcom*:ab,ti
15	reticulosarcom*:ab,ti
16	‘reticulum-cell sarcom*’:ab,ti
17	'hodgkin disease'/exp
18	hodgkin*:ab,ti
19	germinoblastom*:ab,ti
20	reticulolymphosarcom*:ab,ti
21	(malignan* NEAR/2 (lymphogranulom* OR granulom*)):ab,ti
22	OR/1-21
23	rituximab*:ab,ti
24	mabt*:ab,ti
25	ritux*:ab,ti
26	idec*:ab,ti
27	(idec-c2b8 OR idecc2b8):ab,ti
28	gp2013:ab,ti
29	ofatumumab*:ab,ti
30	arzerr*:ab,ti
31	(humax-cd20 OR humaxcd20):ab,ti
32	ocrelizumab*:ab,ti
33	(r-1594 OR r1594):ab,ti
34	pr070769:ab,ti
35	veltuzumab*:ab,ti
36	(immu-106 OR immu106):ab,ti

37	obinutuzumab*:ab,ti
38	gazyva*:ab,ti
39	(r-7159 OR r7159):ab,ti
40	(ro-5072759 OR ro5072759):ab,ti
41	(ga-101 OR ga101):ab,ti
42	ocaratuzumab*:ab,ti
43	ame133:ab,ti
44	(ly2469298 OR ly2469298):ab,ti
45	ublituximab*:ab,ti
46	(tg-1101 OR tg1101):ab,ti
47	pro131921:ab,ti
48	tositumomab*:ab,ti
49	bexxar*:ab,ti
50	(iodine-131 OR I-131 OR 131I):ab,ti
51	(anti-B1 OR anti-CD20):ab,ti
52	50 AND 51
53	ibritumomab*:ab,ti
54	zevalin*:ab,ti
55	(idec-y2b8 OR idecy2b8):ab,ti
56	'cd20 antigens'/exp
57	(CD20 NEAR/3 antibod*) or (CD - 20 NEAR/3 antibod*) or ('CD 20' NEAR/3 antibod*):ab,ti
58	(anti-cd20):ab,ti
59	(anticd20 OR anti-cd-20 OR anticd-20):ab,ti
60	OR/23-49 52-59
61	'randomised controlled trial'/exp OR 'single blind procedure'/exp OR 'double blind procedure'/exp OR 'crossover procedure'/exp
62	random*:ab,ti,tt OR placebo*:ab,ti,tt OR allocat*:ab,ti,tt OR crossover*:ab,ti,tt OR 'cross over':ab,ti,tt OR trial:ti OR (doubl* NEXT/1 blind*):ab,ti,tt
63	61 OR 62
64	'animal'/de OR 'animal experiment'/de OR 'nonhuman'/de
65	'human'/de
66	64 AND 65
67	64 NOT 66
68	63 NOT 67
69	22 and 60 and 68

Cochrane Library:

1	MeSH descriptor Lymphoma explode all trees
2	lymphom* or linfom*
3	MeSH descriptor Hematologic Neoplasms explode all trees
4	(lympho* NEAR/2 (neoplasm* or malign* or tumor* or tumour* or sarcom*))
5	(lympha* NEAR/2 (neoplasm* or malign* or tumor* or tumour* or sarcom*))
6	(hemato* NEAR/1 (malign* or neoplas*))
7	(haemato* NEAR/1 (malign* or neoplas*))
8	MeSH descriptor Lymphoma, Non-Hodgkin explode all trees
9	(nonhodgkin* or non-hodgkin* or non hodgkin* or no hodgkin* or nhl*)
10	mycos* fungo*
11	sezary*
12	granulom*
13	burkitt*
14	lymphosarcom*
15	reticulosarcom*
16	reticulum-cell sarcom*
17	MeSH descriptor Hodgkin Disease explode all trees
18	hodgkin*
19	Germinoblastom*
20	Reticulolymphosarcom*
21	(malignan* NEAR/2 (lymphogranulom* or granulom*))
22	or/1-21
23	rituximab*
24	mabt*
25	ritux*
26	idec*
27	(idec-c2b8 or idecc2b8)
28	gp2013
29	ofatumumab*
30	arzerr*
31	(humax-cd20 or humaxcd20)
32	ocrelizumab*
33	(r-1594 or r1594)
34	pr070769
35	veltuzumab*
36	(immu-106 or immu106)
37	obinutuzumab*

38	gazyva*
39	(r-7159 or r7159)
40	(ro-5072759 or ro5072759)
41	(ga-101 or ga101)
42	ocaratuzumab*
43	ame133
44	(ly2469298 or ly2469298)
45	ublituximab*
46	(tg-1101 or tg1101)
47	pro131921
48	tositumomab*
49	bexxar*
50	(iodine-131 or I-131 or 131I)
51	(anti-B1 or anti-CD20)
52	50 and 51
53	ibritumomab*
54	zevalin*
55	(idec-y2b8 or idecy2b8)
56	MeSH descriptor Antigens, CD20 explode all trees
57	((cd20 or cd-20 or cd 20) NEAR/3 antibod*)
58	(anti-cd20 or anti cd20)
59	(anticd20 or anti-cd-20 or anticd-20)
60	or/23-49 52-59
61	22 and 60

Table S1. Risk of bias for the included studies.

Trial	Sequence generation	Allocation concealment	Blinding of outcome Assessment	Incomplete data	Selective reporting
GAINED	Low	Unclear	Low	Low	Unclear
GALLIUM	Low	Unclear	Low	Low	Low
GAUSS	Low	Unclear	Low	Low	Low
GOYA	Low	Unclear	Low	Low	Low
HOMER	Low	Unclear	Low	Unclear	Low
ORCHARRD	Low	Unclear	Low	Low	Low
BMT CTN 0401	Low	Unclear	Low	Low	Low
Quackenbush 2015	Low	Unclear	Unclear	Low	Low
SWOG S0016	Low	Low	Low	Low	Low
Khouri 2015	Unclear	Unclear	Unclear	Low	Low
Witzig 2002	Low	Unclear	Low	Low	Low

Table S2. Adverse events (Obinutuzumab versus Rituximab).

Name	No. of trials	No. of patients (events/total)		OR (95% CI)	*P*
		Obinutuzumab arm	Rituximab arm		
All AEs	3	1351/1386	1300/1386	2.73 (1.81-4.13)	<0.00001
Grade 3-5 AEs	3	978/1386	868/1386	1.50 (1.26-1.77)	<0.00001
SAEs	4	734/1722	630/1720	1.29 (1.13-1.48)	0.0003
Fatal AEs	3	75/1635	52/1634	1.47 (1.02-2.10)	0.04
Infusion-related reaction (IRR)	3	669/1386	501/1386	1.71 (1.46-2.00)	<0.00001
Grade 3-5 IRR	3	119/1386	50/1386	2.51 (1.79-3.52)	<0.00001
Neutropenia	3	632/1386	553/2386	1.28 (1.09-1.49)	0.002
Grade 3-5 Neutropenia	3	593/1386	500/1386	1.34 (1.15-1.56)	0.0002
Leucopenia	2	184/1299	158/1300	1.17 (0.83-1.66)	0.13
Grade 3-5 Leucopenia	2	147/1299	121/1300	1.24 (0.96-1.60)	0.09
Anemia	2	148/1299	159/1300	0.92 (0.73-1.17)	0.50
Grade 3-5 Anemia	2	75/1299	66/1300	1.14 (0.82-1.57)	0.43
Grade 3-5 Thrombocytopenia	2	67/1299	26/1300	2.67 (1.69-4.23)	<0.0001
Second malignancies	2	44/1299	36/1300	1.23 (0.79-1.93)	0.36
Fatigue	3	374/2386	358/1386	1.06 (0.90-1.27)	0.47
Cough	3	270/1386	223/1386	1.42 (0.95-2.12)	0.09
Pyrexia	3	312/1386	219/1386	1.49 (1.04-2.13)	0.03
Nausea	3	494/1386	483/1386	1.04 (0.89-1.22)	0.64
Asthenia	2	77/791	81/789	0.94 (0.68-1.31)	0.73
Diarrhea	3	279/1386	230/1386	1.21 (1.05-1.55)	0.01
Arthralgia	2	92/682	87/683	1.07 (0.78-1.47)	0.68
Abdominal pain	2	63/682	70/683	0.89 (0.62-1.28)	0.53
Upper respiratory tract infection	2	121/682	116/683	1.06 (0.80-1.40)	0.71
Headache	3	205/1386	165/1386	1.29 (1.03-1.61)	0.02
Decreased appetite	3	174/1386	148/1386	1.24 (0.81-1.90)	0.31
Grade 3-5 Pneumonia	2	68/1299	64/1300	1.07 (0.75-1.51)	0.72
Grade 3-5 Infections	2	256/1299	207/1300	1.30 (1.06-1.59)	0.01
Constipation	2	375/1299	360/1300	1.06 (0.89-1.26)	0.5
Chills	2	232/1299	93/1300	2.89 (1.32-6.07)	0.007
Insomnia	2	162/1299	129/1300	1.30 (1.01-1.66)	0.04
Alopecia	2	225/1299	210/1300	1.09 (0.88-1.34)	0.43
Rash	2	99/682	111/683	0.88 (0.65-1.18)	0.38

AE: adverse event. IRR: infusion-related reaction

Table S3. Subgroup analysis (Obinutuzumab vs Rituximab)

Subgroups	No. of trials	No. of patients		HR/OR (95%CI)	*P* (overall effects)	*P* (subgroup differences)
		Obinutuzumab arm	Rituximab arm			
PFS						
Treatment history						0.63
Previously untreated	2	1307	1313	0.83 (0.72-0.96)	0.01	
Relapsed/Refractory	1	74	75	0.93 (0.60-1.44)	0.75	
Disease subtype						0.18
FL	2	675	676	0.76 (0.63-0.92)	0.006	
DLBCL	1	706	712	0.92 (0.76-1.11)	0.39	
Concomitant chemotherapy regimens						0.32
Bendamustine	1	345	341	0.63 (0.46-0.87)	0.005	
CHOP	2	902	915	0.88 (0.74-1.05)	0.15	
CVP	1	60	57	0.79 (0.42-1.48)	0.46	
None	1	74	75	0.93 (0.60-1.44)	0.75	
Gender						0.08
male	2	652	663	0.97 (0.73-1.31)	0.86	
female	2	620	609	0.62 (0.41-0.94)	0.02	
Race						0.54
Asian	2	364	356	0.89 (0.67-1.17)	0.39	
White	2	913	911	0.80 (0.66-0.97)	0.02	
Bulky disease						0.15
Yes	2	516	533	0.73 (0.58-0.91)	0.007	
No	2	787	777	0.91 (0.74-1.12)	0.38	
Ann Arbor stage						0.28
Stage Ⅰ	2	65	56	0.96 (0.41-2.22)	0.92	
Stage Ⅱ	2	156	167	1.25 (0.75-2.07)	0.40	
Stage Ⅲ	2	419	464	0.71 (0.53-0.94)	0.02	
Stage Ⅳ	2	664	621	0.79 (0.64-0.97)	0.02	
ECOG PS						0.14
0-1	2	1203	1189	0.78 (0.66-0.92)	0.003	
2-3	2	102	122	1.09 (0.71-1.66)	0.69	
IPI						0.11
Low	2	504	534	0.97 (0.75-1.26)	0.81	
Intermediate	2	445	415	0.65 (0.49-0.85)	0.002	
High	2	358	364	0.82 (0.63-1.07)	0.15	
OS						
Treatment history						NA
Previously untreated	2	1307	1313	0.96 (0.78-1.18)	0.70	
Relapsed/Refractory	NA	NA	NA	NA	NA	
Disease subtype						0.57
FL	1	601	601	0.88 (0.61-1.27)	0.49	
DLBCL	1	706	712	1.00 (0.78-1.28)	1.00	
ORR						
Treatment history						0.17
Previously untreated	2	1307	1313	1.12 (0.91-1.38)	0.29	
Relapsed/Refractory	1	88	87	1.77 (0.95-3.28)	0.07	
Disease subtype						0.52

FL	2	675	676	1.25 (0.92-1.70)	0.15	
DLBCL	1	706	712	1.09 (0.84-1.43)	0.50	
Concomitant chemotherapy regimens						0.42
Bendamustine	1	173	165	1.54 (0.87-2.71)	0.14	
CHOP	2	809	815	1.09 (0.84-1.40)	0.52	
CVP	1	21	30	1.25 (0.37-4.21)	0.72	
None	1	88	87	1.77 (0.95-3.28)	0.07	
CRR						
Treatment history						0.09
Previously untreated	2	1307	1313	0.92 (0.67-1.24)	0.57	
Relapsed/Refractory	1	88	87	2.66 (0.80-8.83)	0.11	
Disease subtype						0.85
FL	2	675	676	1.18 (0.40-3.49)	0.77	
DLBCL	1	706	712	1.06 (0.85-1.32)	0.61	
Concomitant chemotherapy regimens						0.40
Bendamustine	1	173	165	1.10 (0.71-1.72	0.65	
CHOP	2	809	815	1.08 (0.88-1.32)	0.46	
CVP	1	21	30	2.00 (0.56-7.15	0.29	
None	1	88	87	2.66 (0.80-8.83)	0.11	

FL, follicular lymphoma; DLBCL, diffuse large B-cell lymphoma; CHOP, cyclophosphamide, doxorubicin, vincristine, and prednisone; CVP, cyclophosphamide, vincristine, and prednisone; Eastern Cooperative Oncology Group performance status: ECOG PS; IPI: international prognostic index.

Table S4. Adverse events (Ofatumumab versus Rituximab).

Name	No. of trials	No. of patients (events/total)		OR (95% CI)	*P*
		Ofatumumab	Rituximab		
Infusion related AEs	1	178/205	118/204	4.80 (2.94-7.85)	<0.00001
Grade 3-5 Infusion related AEs	1	45/205	10/204	5.46 (2.67-11.17)	<0.00001
Gastrointestinal disorders	1	78/205	55/204	1.66 (1.09-2.53)	0.02
Neutropenia	1	6/205	14/204	0.41 (0.15-1.09)	0.07
Pyrexia	1	8/205	20/204	0.37 (0.16-0.87)	0.02
AEs leading to dose interruptions	1	109/222	67/223	2.25 (1.52-3.31)	<0.00001
Grade 4 thrombocytopenia	1	78/222	78/223	1.01 (0.68-1.49)	0.97
Grade 4 neutropenia	1	49/222	58/223	0.81 (0.52-1.25)	0.33
Rash	1	49/222	20/223	2.87 (1.65-5.02)	0.0002
Raised serum creatinine	1	51/222	36/223	1.55 (0.96-2.49)	0.07

Table S5. Subgroup analysis (Ofatumumab vs Rituximab)

Subgroups	No. of trials	No. of patients		HR/OR (95%CI)	*P* (overall effects)	*P* (subgroup differences)
		Ofatumumab arm	Rituximab arm			
PFS						
Treatment history						NA
Previously untreated	NA	NA	NA	NA	NA	
Relapsed/Refractory	2	427	427	1.13 (0.95-1.34)	0.17	
Disease subtype						0.92
FL	1	205	204	1.14 (0.88-1.47)	0.31	
DLBCL	1	222	223	1.12 (0.89-1.41)	0.34	
Concomitant chemotherapy regimens						0.92
None	1	205	204	1.14 (0.88-1.47)	0.31	
DHAP	1	222	223	1.12 (0.89-1.41)	0.34	
OS						
Treatment history						NA
Previously untreated	NA	NA	NA	NA	NA	
Relapsed/Refractory	2	427	427	0.93 (0.74-1.17)	0.54	
Disease subtype						0.45
FL	1	205	204	1.18 (0.61-2.29)	0.62	
DLBCL	1	222	223	0.90 (0.70-1.15)	0.41	
Concomitant chemotherapy regimens						0.45
None	1	205	204	1.18 (0.61-2.29)	0.62	
DHAP	1	222	223	0.90 (0.70-1.15)	0.41	
ORR						
Treatment history						NA
Previously untreated	NA	NA	NA	NA	NA	
Relapsed/Refractory	2	180/427	213/427	0.73 (0.55-0.96)	0.02	
Disease subtype						0.31
FL	1	205	204	0.63 (0.43-0.93)	0.02	
DLBCL	1	222	223	0.84 (0.57-1.22)	0.35	
Concomitant chemotherapy regimens						0.31
None	1	205	204	0.63 (0.43-0.93)	0.02	
DHAP	1	222	223	0.84 (0.57-1.22)	0.35	
CRR						
Treatment history						
Previously untreated	NA	NA	NA	NA	NA	
Relapsed/Refractory	1	34/222	48/223	0.66 (0.41-1.07)	0.09	
Disease subtype						NA
FL	1	205	204	NA	NA	
DLBCL	1	222	223	0.66 (0.41-1.07)	0.09	
Concomitant chemotherapy regimens						NA
None	1	205	204	NA	NA	
DHAP	1	222	223	0.66 (0.41-1.07)	0.09	

FL, follicular lymphoma; DLBCL, diffuse large B-cell lymphoma; DHAP, cisplatin, cytarabine, dexamethasone.

Table S6. Adverse events (Tositumomab versus Rituximab).

Name	No. of trials	No. of patients (events/total)		OR (95% CI)	*P*
		Tositumomab	Rituximab		
Grade 3-5 AEs	1	67/103	46/107	2.47 (1.41-4.31)	0.001
Grade 3-5 Neutropenia	2	136/271	127/269	1.14 (0.81-1.60)	0.45
Grade 3-5 Thrombocytopenia	2	49/271	6/269	9.03 (3.90-20.89)	<0.00001
Grade 3-5 Anemia	1	8/263	7/263	1.15 (0.41-3.21)	0.79
Grade 3-5 Infection	1	45/263	61/263	0.68 (0.44-1.05)	0.08
Grade 3-5 Febrile neutropenia	1	26/263	42/263	0.58 (0.34-0.97)	0.04
Grade 3-5 Fatigue	1	9/263	11/263	0.81 (0.33-1.99)	0.65
Second Malignancies	2	41/366	45/370	0.90 (0.57-1.42)	0.64
Cardiovascular events	2	20/366	32/370	0.61 (0.34-1.09)	0.10
Gastrointestinal events	2	33/366/	34/370	0.98 (0.59-1.62)	0.94
Grade 3-5 Hypoxia	1	20/103	17/107	1.28 (0.63-2.60)	0.50
Grade 3-5 Dyspnea	1	29/103	25/107	1.29 (0.69-2.39)	0.43
Grade 3-5 Mucositis	1	53/103	19/107	4.91 (2.62-9.21)	<0.00001
Grade 1-2 thyroid dysfunction	1	18/263	8/263	2.34 (1.00-5.49)	0.05
HAMA formation	1	47/263	41/263	1.18 (0.74-1.86)	0.48

HAMA: human antimouse antibody

Table S7. Subgroup analysis (Tositumomab vs Rituximab)

Subgroups	No. of trials	No. of patients		HR/OR (95%CI)	*P* (overall effects)	*P* (subgroup differences)
		Tositumomab arm	Rituximab arm			
PFS						
Treatment history						0.88
Previously untreated	1	264	267	0.73 (0.58-0.93)	0.01	
Relapsed/Refractory	2	119	119	0.66 (0.19-2.27)	0.52	
Disease subtype						0.16
FL	2	272	273	0.59 (0.28-1.26)	0.17	
DLBCL	1	111	113	1.08 (0.76-1.54)	0.67	
Concomitant chemotherapy regimens						0.07
CHOP	1	264	267	0.73 (0.58-0.93)	0.01	
BEAM	1	111	113	1.08 (0.76-1.54	0.67	
None	1	8	6	0.30 (0.08-1.11)	0.07	
OS						
Treatment history						0.35
Previously untreated	1	264	267	1.33 (0.93-1.92)	0.12	
Relapsed/Refractory	2	119	119	0.37 (0.03-5.10)	0.46	
Disease subtype						0.47
FL	1	272	273	0.40 (0.03-6.40)	0.52	
DLBCL	1	111	113	1.14 (0.73-1.78)	0.56	
Concomitant chemotherapy regimens						0.05
CHOP	1	264	267	1.33 (0.93-1.92)	0.12	
BEAM	1	111	113	1.14 (0.73-1.78)	0.56	
None	1	8	6	0.08 (0.01-0.75)	0.03	
ORR						
Treatment history						0.16
Previously untreated	1	264	267	0.79 (0.21-2.97)	0.72	
Relapsed/Refractory	1	8	6	9.44 (0.37-242.18)	0.17	
Disease subtype						NA
FL	2	272	273	1.25 (0.40-3.93)	0.70	
DLBCL	1	111	113	NA	NA	
Concomitant chemotherapy regimens						0.16
CHOP	1	264	267	0.79 (0.21-2.97)	0.72	
BEAM	1	111	113	NA	NA	
None	1	8	6	9.44 (0.37-242.18)	0.17	
CRR						
Treatment history						0.06
Previously untreated	1	264	267	1.17 (0.83-1.67)	0.37	
Relapsed/Refractory	1	8	6	15.00 (1.03-218.30)	0.05	
Disease subtype						NA
FL	2	272	273	2.93 (0.27-32.18)	0.38	
DLBCL	NA	NA	NA	NA	NA	
Concomitant chemotherapy regimens						0.06
CHOP	1	264	267	1.17 (0.83-1.67)	0.37	
BEAM	1	111	113	NA	NA	
None	1	8	6	15.00 (1.03-218.30)	0.05	

FL, follicular lymphoma; DLBCL, diffuse large B-cell lymphoma; CHOP, cyclophosphamide, doxorubicin, vincristine, and prednisone; BEAM: carmustine, etoposide, cytarabine, and melphalan.

Table S8. Adverse events (Ibritumomab versus Rituximab).

Name	No. of trials	No. of patients (events/total)		OR (95% CI)	*P*
		Ibritumomab	Rituximab		
Asthenia	1	35/73	28/70	1.38 (0.71-2.68)	0.34
Nausea	1	31/73	13/70	3.24 (1.51-6.92)	0.002
Chills	1	18/73	20/70	0.82 (0.39-1.72)	0.60
Pain	1	15/73	11/70	1.39 (0.59-3.27)	0.45
Fever	1	14/73	12/70	1.15 (0.49-2.69)	0.75
Abdominal pain	1	14/73	8/70	1.84 (0.72-4.70)	0.20
Vomiting	1	14/73	5/70	3.08 (1.05-9.09)	0.04
Throat irritating	1	13/73	11/70	1.16 (0.48-2.80)	0.74
Headache	1	12/73	16/70	0.66 (0.29-1.53)	0.34
Increased cough	1	11/73	5/70	2.31 (0.76-7.02)	0.14
Dizziness	1	11/73	5/70	2.11 (0.77-5.76)	0.14
Dyspnea	1	11/73	5/70	2.31 (0.76-7.02)	0.14
Pruritus	1	8/73	12/70	0.59 (0.23-1.56)	0.29
Angioedema	1	6/73	11/70	0.48 (0.17-1.38)	0.17
